# Therapeutic Potential of Hematopoietic Prostaglandin D_2_ Synthase in Allergic Inflammation

**DOI:** 10.3390/cells8060619

**Published:** 2019-06-20

**Authors:** Sonja Rittchen, Akos Heinemann

**Affiliations:** 1 Otto Loewi Research Center for Vascular Biology, Immunology and Inflammation, Division of Pharmacology, Medical University of Graz, Graz 8010, Austria; sonja.rittchen@medunigraz.at; 2 BioTechMed, Graz 8010, Austria

**Keywords:** hPGDS, hPGDS inhibitor, PGD_2_, DP receptors, allergic inflammation, eosinophilic inflammation

## Abstract

Worldwide, there is a rise in the prevalence of allergic diseases, and novel efficient therapeutic approaches are still needed to alleviate disease burden. Prostaglandin D_2_ (PGD_2_) has emerged as a central inflammatory lipid mediator associated with increased migration, activation and survival of leukocytes in various allergy-associated disorders. In the periphery, the hematopoietic PGD synthase (hPGDS) acts downstream of the arachidonic acid/COX pathway catalysing the isomerisation of PGH_2_ to PGD_2_, which makes it an interesting target to treat allergic inflammation. Although much effort has been put into developing efficient hPGDS inhibitors, no compound has made it to the market yet, which indicates that more light needs to be shed on potential PGD_2_ sources and targets to determine which particular condition and patient will benefit most and thereby improve therapeutic efficacy. In this review, we want to revisit current knowledge about hPGDS function, expression in allergy-associated cell types and their contribution to PGD_2_ levels as well as beneficial effects of hPGDS inhibition in allergic asthma, rhinitis, atopic dermatitis, food allergy, gastrointestinal allergic disorders and anaphylaxis.

## 1. Introduction

**Figure 1:**
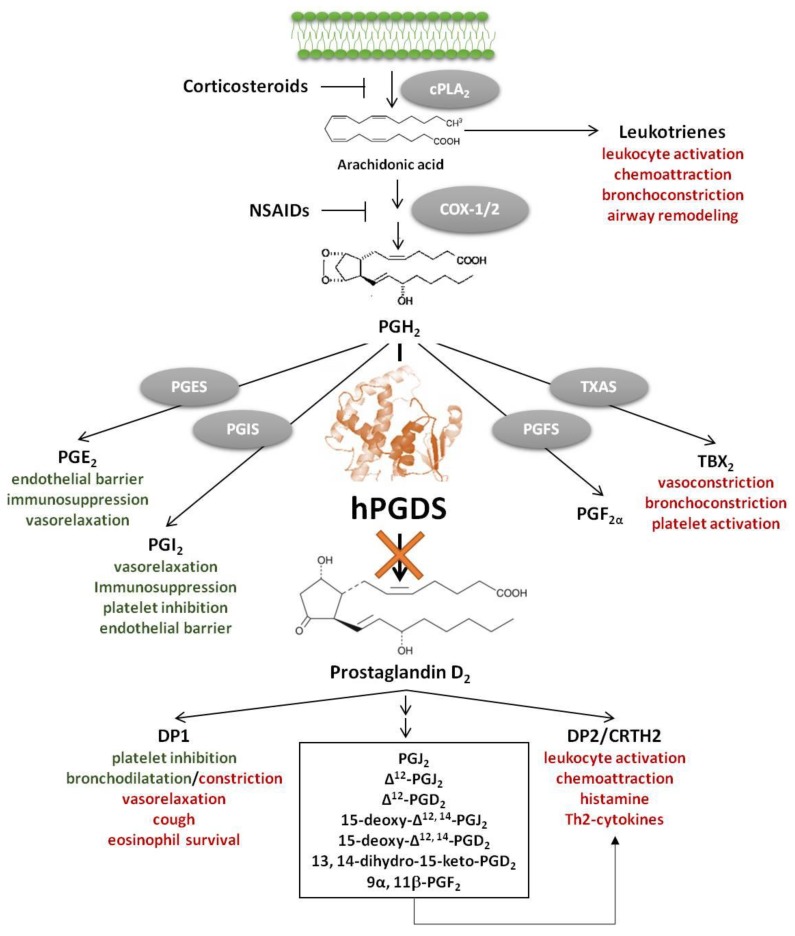
hPGDS as therapeutic target downstream of the arachidonic acid/cyclooxygenase (COX) pathway. Hematopoietic PGDS inhibition specifically targets PGD_2_ and PGD_2_ metabolite production—mediators that primarily activate pro-inflammatory DP2/CRTH2 receptor [1]. Non-steroidal anti-inflammatory drugs (NSAIDs) block all lipid mediators downstream of COX-1/2, including potentially beneficial effects of PGE_2_ and PGI_2_. Corticosteroids are standard-of-care therapeutics of asthmatic patients that effectively block all downstream products of arachidonic acid including leukotrienes; however, therapy interferes with many physiological processes causing numerous adverse effects. Favorable effects of selected lipid mediators in allergic inflammation highlighted in green; unfavorable effects highlighted in red.

Accumulating evidence suggests a central role of the pro-inflammatory lipid mediator Prostaglandin D_2_ (PGD_2_) in allergy development and progression [1,2,3]. PGD_2_ is a potent pro-inflammatory lipid mediator downstream of the arachidonic acid/cyclooxygenase (COX) pathway. Arachidonic acid-derived lipid mediators including leukotrienes, lipoxins, thromboxane A_2_, PGD_2_, prostaglandin E_2_ (PGE_2_) and prostacyclin (PGI_2_) play a central role in allergic inflammation; each of them having specific immunomodulatory functions (Figure 1). Notably, in contrast to COX inhibition, specific inhibition of unfavourable pro-inflammatory PGD_2_ effects and its metabolites would keep physiological functions of beneficial mediators like PGE_2_ and prostacyclin intact. In mice, about 90% of the systemic biosynthesis of PGD_2_ is generated by the hematopoietic PGD synthase (hPGDS)-dependent pathway and only partially through lipocalin-type PGD synthase (LPGDS) [4]. Most prostaglandins are generated by competitive enzymatic interactions, however, it has been suggested that prostaglandins may also be generated from precursor eicosanoids by non-enzymatic conversion [5], which also needs to be taken into account in a therapeutic setting. PGD_2_ exerts its function by activating two G-protein coupled receptors, d-type prostanoid receptor 1 (DP1) and 2 (DP2), the latter also being referred to as chemoattractant receptor homologous-molecule expressed in Th2 cells (CRTH2) [6]. DP1-mediated responses include inhibition of platelet aggregation, vasorelaxation and bronchodilatation [7], but DP1 antagonists have also been found to ameliorate rhinitis, conjunctivitis and pulmonary inflammation in animal models [8,9,10], while DP1 receptor activation aggravated neutrophil infiltration in acute lung injury [11]. In contrast, DP2/CRTH2 receptor activation has primarily been linked to pro-inflammatory effects including initiation and potentiation of immune cell migration, respiratory burst, type 2 cytokine production and histamine release [3]. PGD_2_ is a potent modulator of inflammation; apparently, its influence strongly depends on whether it acts in the early or late phase of inflammation. On the one hand, it has been reported that in acute inflammation, i.e., experimental dermatitis [12] and colitis [13], lipopolysaccharide-induced pulmonary inflammation [14] as well as in anaphylactic shock [15], PGD_2_ seems to have protective effects. On the other hand, in late phase skin inflammation [12], and chronic and allergic inflammation [16,17], PGD_2_/CRTH2/DP2 activation exacerbates leukocyte migration, activation and survival, while DP1 activation has been linked to increased mucus production and airway hyperreactivity [18]. In addition, some PGD_2_ metabolites, such as 15-deoxy-Δ^12,14^-PGJ_2_ have been shown to exert anti-inflammatory, pro-resolving effects by activating nuclear receptors, e.g., peroxisome proliferator-activated receptors (PPAR)-γ [19] but the physiological relevance thereof is still unclear [20]. 

Taken together, both PGD_2_ receptors, DP1 and DP2/CRTH2, have emerged as potential drug targets for the treatment of allergic diseases and beyond [1,21,22]. However, as an alternative to receptor blockade, great clinical interest has also been attributed to the development of hPGDS inhibitors to nip PGD_2_ signalling in the bud and thereby attenuate allergic inflammation, and potentially other conditions. 

## 2. hPGDS Structure, Function and Regulation

Two distinct rate-limiting PGD synthases have been described, lipocalin-type PGD synthase (LPGDS) and hematopoietic PGD synthase (hPGDS), which differ vastly in origin, structure, tissue distribution, and functional context. LPGDS is primarily localized in the central nervous system, and reproductive tracts; it is secreted into cerebrospinal fluid and the bloodstream, whereby this enzyme does not need reduced glutathione (GSH) as a co-factor [23]. In contrast, hPGDS is a Sigma-class glutathione transferase expressed in peripheral tissues and catalyzes the isomerization of PGH_2_ to PGD_2_ using GSH and Ca^2+^ or Mg^2+^ as cofactors [24]. The hPGDS enzyme forms a homodimer with 23 kDa subunits and each subunit is associated with one GSH [25]. Site-directed mutagenesis indicates that Lys112, Cys156, and Lys198 are involved in the binding of PGH_2_, Trp104 is critical for structural integrity of the catalytic center for GSH-transferase (GST) and PGD synthase activities, and Tyr8 and Arg14 are essential for activation of the thiol group of glutathione [26]. Notably, LPGDS is quite different in terms of catalytic properties, amino acid sequence, tertiary structure, evolutional origin, gene structure, chromosomal localization, cellular localization, tissue distribution and also functional relevance—a good example of functional convergence [23]. A number of naturally occurring hPGDS enzyme variants have been identified, differing in thermal stability and half-life in relation with GST-activity [27]. In this study, they could identify a highly stable hPGDS isoenzyme (Val187Ile) found in African Americans, which seems to be associated with reduced colorectal cancer risk. However, whether different hPGDS variants are associated with allergic disease or other inflammatory disorders has not been clarified to date. Aritake et al. suggest a functional coupling between COX-2 and hPGDS as well as a possible role of hPGDS membrane translocation in modulation of efficient PGD_2_ synthesis [28]. There is evidence that hPGDS activity is dependent on pH, which has an impact on H+ abstraction from the GSH thiol group [24]. Zhao et al. could show that reactive oxygen species are crucial for proper hPGDS function. Selective inhibition of NADPH oxidase-2 in murine bone marrow-derived macrophages attenuated bacterial lipopolysaccharide (LPS)-induced production of PGD_2_ but not PGE_2_ [29]. In line with this, Ishii highlights the importance of the transcription factor nuclear-erythroid-2 p45-related factor (Nrf2) in combination with peroxiredoxin 1 and 6 in regulating PGD_2_ production by hPGDS in murine bone marrow-derived macrophages [30]. Oxidative stress and activation of the Toll-like receptor 4 trigger nuclear factor-кB signaling and peroxiredoxin 6 phosphorylation, a bifunctional enzyme with glutathione peroxidase and phospholipase A_2_ activity, which in turn activates NADPH Oxidase-2 [31]. This also stresses the importance of NADPH oxidase-2 activation in maintaining cellular glutathione levels to ensure efficient hPGDS function. Interestingly, Nrf2 seems to be involved in a positive feedback induction of LPGDS by hPGDS-derived PGD_2_ and 15-deoxy-Δ12,14-PGJ_2_ in murine macrophages, thereby contributing to resolution of LPS-induced lung inflammation in mice [32]. Another study in human eosinophils found that PGD_2_ synthesis was located at the nuclear envelope and was associated with intracellular lipid bodies [33]. Inhibition of hPGDS with a specific inhibitor (HQL-79) also reduced lipid body formation in eosinophils. 

## 3. Hematopoietic PGDS Expression in Leukocytes and Parenchymal Cells

Hematopoietic PGD synthase is differentially expressed in peripheral tissues, varying between species. In the rat, hPGDS could be detected on protein level in spleen, bone marrow, liver, colon, small intestine, skin and thymus, while the highest expression of hPGDS mRNA in human tissue was found in macrophages, placenta, intestine, adipose tissue and foetal liver [34]. Allergy-associated cell types reported to express hPGDS on mRNA or protein level and their PGD2 production potential have been summarized in Table 1. 

### 3.1. Immune Cells

Mast cells have been characterized most thoroughly as potential PGD_2_ sources in allergic disease. They are mainly tissue-resident and located in the skin and mucosal surfaces throughout the body, with increased abundance in asthmatic patients, inflammatory bowel disease and atopic dermatitis [35,36,37]. A transcriptional signature approach revealed that the hPGDS transcript is enriched in human and murine tissue-resident mast cells as compared to other cells analyzed [38]. Upon allergen-induced cross-linking of Fcε-bound IgE mast cells produce high local concentrations of PGD_2_ in the nanomolar range [39]. Baothman et al. observed that PGD2 generation in isolated human lung mast cells largely relied on COX-1 and hPGDS but not LPGDS, while COX-2 was absent both at baseline and after LPS or stem cell factor stimulation [40]. In food antigen-induced mast cell hyperplasia in mice, c-Kit^+^/FcE-RI^+^ mast cells in colon sections stained highly positive for hPGDS, and tetranor PGD metabolite, a mast cell-derived PGD_2_ metabolite, proved to be a specific biomarker for food allergy in patients [41,42]. Higher levels of hPGDS but not LPGDS expression could also be found in the nasal mucosa of patients with allergic rhinitis, where mast cells could be identified as hPGDS^+^ cells next to infiltrating eosinophils, macrophages and lymphocytes [43]. In mice, hPGDS^+^ lung tumor-infiltrating c-Kit^+^/FcE-RI^+^ mast cells could be identified by immunofluorescence microscopy [40,44]. 

Granulocytes are short-lived cells of the innate immune system with characteristically lobed nuclei and carry granules with antimicrobial peptides and cytotoxic proteins. Accumulation of eosinophil granulocytes is a hallmark of allergic inflammation where they contribute to airway hyper-reactivity in asthma, mucus production and tissue remodeling [45,46]. Activated eosinophils secrete various cytotoxic proteins and pro-inflammatory mediators that drive Th2-type inflammation including IL-4, IL-5, IL-10 and IL-13 [47]. Both DP receptors are expressed on the cell surface, mediating eosinophil migration and activation by PGD_2_ [3,48,49]; however, recently it has been shown that eosinophils themselves can act as PGD_2_ sources. Human peripheral blood eosinophils and mouse bone marrow-derived eosinophils constitutively express hPGDS and release up to 80 pg PGD_2_/ 2 × 10^6^ cells upon stimulation in a HQL-79-sensitive manner [33]. Similarly, infiltrating eosinophils in the nasal mucosa of patients with allergic rhinitis express hPGDS [43]. Interestingly, Feng et al. observed that human peripheral blood eosinophils from patients with aspirin-exacerbated respiratory disease express significantly higher levels of hPGDS on protein and mRNA level than eosinophils from asthmatic or healthy subjects [50]. Eosinophils from these patients released PGD_2_ up to 1 ng/10^5^ cells upon stimulation. 

Basophils, despite their relatively low numbers in comparison with other effector cells, such as mast cells and eosinophils, have attracted notice in allergic responses including systemic anaphylaxis, atopic dermatitis, allergic rhinitis and asthma [51,52,53,54,55]. Via DP2/CRTH2, PGD_2_ acts as potent chemoattractant and activator for basophils which may result in basophil accumulation at sites of inflammation [2]. Like mast cells, basophils are activated by IgE-receptor cross-linking, which triggers secretion of various cytokines favoring a type-2 inflammation, including IL-4 [51]. Besides contributing to cytokine levels in allergic inflammation, basophils have also been considered as PGD_2_ sources. Ugajin et al. showed that murine basophils express hPGDS on mRNA and protein level. Further, sensitization of mouse bone marrow-derived as well as peripheral blood basophils with anti-TNP-IgE and subsequent stimulation with TNP-OVA triggered PGD_2_ (up to 700 pg PGD_2_/5 × 10^5^ basophils) and PGE_2_ release at similar levels [56]. Primary human basophils also released PGD_2_ (100 pg PGD_2_/5 × 10^5^ basophils) after priming with IL-3, sensitization and stimulation with anti-human IgE, but interestingly no PGE_2_ could be detected. The authors suggest that human basophils might favor the hPGDS pathway over the PGES pathway, thereby augmenting allergic inflammation [56]. Recently, another group addressed the role of basophils in systemic lupus erythematosus development and found that autocrine PGD_2_ production and CXCR4-dependent stimulation of basophils can be reversed by using a specific hPGDS inhibitor (HPGDS inhibitor I, Cayman) [57]. 

Neutrophils comprise the most abundant cell population of peripheral blood leukocytes and play a central role in acute bacterial infections and type-1 inflammatory reactions [58]. In addition, increased numbers of activated neutrophils in nasal mucosa of allergic rhinitis patients exhibit T-cell priming capacity and facilitate eosinophil migration [59]. Polak et al. elaborated on this and could show that neutrophil phagocytosis and antigen presentation fully activates allergen-specific T-cells in Birch pollen allergy [60,61]. In the context of PGD_2_, there is evidence of an indirect link between increased PGD_2_ levels and neutrophil influx mediated by macrophages as neutrophils are not directly attracted or activated by PGD_2_ [11]. Hematopoietic PGDS expression in neutrophils has been detected in a murine model of LPS-induced lung inflammation [14], however, PGD_2_ production by neutrophils has not been described to date.

Mononuclear phagocytes are a heterogeneous population of antigen presenting cells with specific, but partly overlapping functions: monocytes primarily serve as precursor cells and cytokine sources, macrophages are highly phagocytic, while dendritic cells are specialized on antigen presentation and activating naïve T-cells [62]. In 1989, Urade et al. reported that antigen-presenting cells, i.e., hPGDS-expressing macrophages and dendritic cells in spleen, thymus, Peyer’s patch of the intestine, submucosal layer of the stomach and in the liver, are likely the major source of PGD_2_ in the rat [63]. 

Peripheral blood monocytes are recruited from the blood to inflamed tissue, where they either actively contribute to inflammation or differentiate into monocyte-derived macrophages and dendritic cells [64]. Challenge of selected patients with inhaled allergens caused changes in circulating monocyte populations 24 h post stimulation [65]. Further, infants who developed food allergy within the first year of life displayed higher number of monocytes and reduced numbers of Treg cells in chord blood, which might contribute to Th2-type inflammation [66]. Human monocytes express both DP1 and DP2/CRTH2 receptors but show only minor response to activation [11]. A study looking into changes in prostaglandin production of human monocytes if cultured in vitro reported a release of PGD_2_ in femtomol-range by unstimulated monocytes, suggesting that those cells should have the machinery to produce PGD_2_ if activated accordingly [67]. In current literature, monocytes have not been considered as PGD_2_ sources to date. 

Macrophages are distributed throughout the body and play a crucial role in maintaining tissue homeostasis, linking innate and adaptive immune response and are exposed continuously to various kinds of particles, toxins, allergens and infectious agents [68]. Most adult tissue macrophages originate from embryonic cells rather than from circulating monocytes, but during inflammation monocytes are recruited to inflamed tissue and act as precursors for monocyte-derived macrophages [69]. A central role of macrophages as mediators of inflammation has been reviewed vastly in allergic asthma [70], food allergy [71] and atopic dermatitis [72]. Human macrophages express both DP receptors and activation with PGD_2_ triggers a chemotactic response as well as release of several inflammatory mediators [11]. Numerous studies showed that murine bone marrow-derived macrophages express hPGDS and release up to 20 ng PGD_2_/mL (1 × 10^6^ cells) upon pro-inflammatory stimuli like LPS or Zymosan A [29,73,74,75]. Further, hPGDS expression in macrophages was reported in the nasal mucosa of allergic rhinitis patients [43], pulmonary macrophages in acute respiratory distress syndrome [11] and human adipose tissue macrophages [76]. One study showed that hPGDS knock-down with specific siRNA but not LPGDS siRNA as well as co-treatment with HQL-79, a hPGDS inhibitor, but not AT-56, a LPGDS inhibitor, attenuated PGD_2_ production by mouse bone marrow-derived macrophages [29]. Recently, Henkel et al. stressed the importance of altered macrophage function in allergic disease by showing that human monocyte-derived macrophages co-stimulated with IL-4 and house dust mite allergen produce PGD_2_ [77]. 

Dendritic cells are highly specified in antigen sampling, processing and presentation to memory and naïve T-cells and they form a strong network of surveillance at sites of antigen entrance such as epithelial and mucosal linings of skin, alimentary and urogenital tract, and airways [78]. Their function is to keep the balance between host defense and tolerance; however, if an allergen is recognized as potentially harmful it is processed and presented to naïve T-cells in lymph nodes leading potentially to a Th2 type inflammatory reaction. PGD_2_ seems to impact the differentiation and function of human monocyte-derived dendritic cells by affecting antigen processing and presentation which leads to a bias in T-cell maturation towards Th2 cells [79]. This effect was seen to be even more pronounced with a DP1-specific agonist. Another study suggested that elevated PGD_2_ levels in neonates, e.g., during respiratory viral infections, cause a delay in dendritic cell maturation and migration to lymph nodes by DP1 activation [80]. As prototype of an antigen presenting cell, dendritic cells have been considered as potential PGD_2_ source as immunohistochemical staining could confirm that these cells express hPGDS [63]. Interestingly, Lee et al. found a relationship between decreased cAMP and increased hPGDS mRNA levels in murine DCs, and in turn a Th2-biased response [81]. Although PGD_2_ production by mouse bone marrow-derived DCs was not measured, the Th2-bias to the immune response was blunted after transfection of these cells with hPGDS-specific siRNA. Another study found that peripheral blood DCs as well as tissue plasmacytoid and myeloid DCs in atopic dermatitis patients stained positive for hPGDS [82]. They could see a PGD_2_ release up to 250 pg/ 1 × 10^6^ human monocyte-derived DCs after LPS stimulation, while LPS caused a downregulation of hPGDS expression after 6 h. Therefore, differences between specific DC populations need to be considered as well as effects due to culture conditions. Accordingly, primary synovial fluid DCs express much higher hPGDS levels than monocyte-derived DCs, but hPGDS expression decreases in synovial fluid DCs after two days in culture [83]. 

Lymphocytes play an important role during induction and maintenance of allergic inflammation. In this context, a dysregulation of the adaptive immune response is initiated which leads to the production of allergen-specific IgE antibodies. It has been reported that PGD_2_ signaling primarily influences Th2 and group 2 innate lymphoid cell (ILC2) function during allergic reactions [54,84]. 

Th2 cells have multiple roles in the initiation and maintenance of allergic inflammation including induction of allergen-specific IgE antibody production, type-2 cytokine secretion and promotion of eosinophilic inflammation [85]. The DP2/CRTH2 receptor has been characterized first in Th2 cells which resulted in the synonym chemoattractant receptor homologous molecule expressed on Th2 cells (CRTH2) and stresses the importance of PGD_2_ for these cells [54]. Increased numbers of DP2/CRTH2^+^CD4^+^ Th2 cells could be found in the circulation and skin lesions of atopic dermatitis patients, and transcriptional profiling of Th2 cells from asthmatic patients also revealed several changes in allergy-associated features including genes relevant for prolonged survival and activation [86,87]. Another study demonstrated that inhibition of PGD_2_ production with an hPGDS inhibitor (HQL-79) as well as blocking DP2/CRTH2 reduced lymphocyte influx in hapten-specific IgE-induced ear swelling [16]. Stimulation of hPGDS^+^DP2/CRTH2^+^ Th2 cells from healthy adults with a combination of OKT3 (monoclonal anti-CD3 antibody) and KOLT-2 (monoclonal anti-CD28 antibody) induced up to 30 ng PGD_2_/mL (5 × 10^6^ cells) [88]. Unlike Th1 cells, hPGDS^+^CRTH2^+^ Th2 cells express hPGDS, which was confirmed by flow cytometry, Northern and Western blotting. Further, Mitson-Salazar et al. could identify hPGDS and DP2/CRTH2 double-positive cells as pro-eosinophilic effector Th2 cell population in patients with eosinophilic gastrointestinal disorder [89]. CD161^+^/hPGDS^+^ Th2 cells release higher levels of IL-5 and IL-13, thereby favoring a type-2 inflammation. 

ILC2s can be found in the lung, skin and gut and are believed to play an important role in development of type 2 inflammation [90]. Comparable to Th2 cells, PGD_2_ regulates the function, migration and accumulation of ILC2s during pulmonary inflammation and is now established as one of the cell surface markers for this lymphoid cell population [91]. Recently, we demonstrated that of autocrine PGD_2_ to enable ILC2 function and activation [84]. We found that human ILC2s express hPGDS on mRNA level and release up to 1.5 ng/mL PGD_2_ (5 × 10^5^ cells) after stimulation with IL-33, IL-25 and TSLP. Disruption of endogenous PGD_2_ production with a COX-1/2 or hPGDS inhibitor (KMN-698, 100 nM) as well as blockade of DP2/CRTH2 reduced IL-5 and IL-13 secretion, CD25 upregulation and PGD_2_ production. 

### 3.2. Parenchymal Cells

Epithelial cells align at the surface of various parts of the body including skin, gastrointestinal and respiratory tract where they form a tight barrier to keep out pathogens as well as allergens. Beyond maintaining their barrier function, epithelial cells at mucosal surfaces are now considered to actively participate in the development and progression of allergic disease [92]. In a neonatal mouse model of viral bronchiolitis it could be shown that bronchial epithelial cells constitutively express low levels of hPGDS, while viral infection in combination with cockroach allergen-sensitization triggered the upregulation of hPGDS expression and PGD_2_ production 10 days post infection [93]. In the same study they could prove that also human bronchial epithelial cells express hPGDS and release up to 1500 pg PGD_2_/mL 24 h after viral infection. In line with this, Jakiela et al. documented changes in the lipidomic profile of rhinovirus-infected airway epithelial cells including an increase in PGD_2_ release [94]. 

The gut epithelium consists of several highly specified cell types while tuft cells are the only cells that constitutively express all enzymes necessary for PGD_2_ synthesis including hPGDS [95]. The role of tuft cells in diseases is still unclear; however, an increase in tuft cell numbers was associated with gastric inflammation as well as various respiratory inflammatory syndromes [95]. In atopic dermatitis, epidermal keratinocytes have been linked to drive Th2-mediated inflammation by contributing to increased TSLP levels [96]. A combination of the viral mimic poly[I:C] and IL-4 triggered the release of TSLP but also LPGDS-derived PGD_2_ by human keratinocytes [97]. In this study, however, they claim that an increase in PGD_2_ or more specifically the PGD_2_-metabolite 15d-PGJ_2_ was able to decrease keratinocyte-derived TSLP. In total, there seems to be a strong connection between epithelial-derived PGD_2_ and viral infections, which could also be important in the development of allergic inflammation.

Endothelial cells line the inner surface of blood vessels and form an active barrier to selectively control extravasation of blood components and circulating cells. Prostaglandins are able to modulate vascular permeability, which needs to be tightly controlled to prevent excessive inflammation [98]. Besides keeping a tight barrier, endothelial cells are part of the innate response to allergens and there is more and more evidence that angiogenesis may be an early step in asthma initiation [99,100]. No studies to date could prove that endothelial cells express hPGDS; however, some studies presented evidence that they express LPGDS and are able to produce PGD_2_ [101,102]. A recently published article showed that LPGDS knock-out mice but not hPGDS knock-out mice display a cardiovascular phenotype. In the vasculature, an autocrine LPGDS-derived PGD_2_ effect seems crucial to protect from hypertension and thrombogenesis [4]. 

Smooth muscle cell hyper-contraction is a characteristic feature of asthmatic airways causing a limitation of gas exchange. In a mouse model of OVA-induced allergic pulmonary inflammation, the COX-2/hPGDS/PGD_2_ cascade was suggested to be involved in the development of bronchial smooth muscle cell hyper-responsiveness [103]. Microarray, RT-qPCR and Western blot results of this study revealed an increased hPGDS expression in bronchial epithelial cells of allergen-challenged mice suggesting an autocrine pathway. Additionally, DP2/CRTH2 activation promotes smooth muscle cell proliferation, thereby contributing to airway remodeling [104].

## 4. PGD2 Signaling as Therapeutic Target in Allergic Diseases

### 4.1. Allergic Asthma and Rhinitis

Asthma is a chronic respiratory condition characterized by airway hyper-responsiveness and airflow obstruction. Despite the availability of reasonably good standard-of-care therapy for most asthmatics, new drugs are needed for severe and poorly controlled asthma to effectively replace oral corticosteroids [108]. PGD_2_ is found in human airways during acute allergen challenge and in patients with severe asthma. Further, disease severity has been shown to correlate with PGD_2_ levels [109,110]. PGD_2_ acts as a chemoattractant and activator of Th2 cells, eosinophils and basophils, primarily initiated by DP2/CRTH2 activation [54]. The role of DP1 in asthma remains elusive; however, in preclinical models of asthma and acute lung injury, DP1 agonism attenuated type 2 inflammation and endothelial cell damage, respectively [8,14]. In contrast, concentrations of PGD_2_ and the DP1 agonist BW245c in the micromolar range trigger smooth muscle contraction and bronchoconstriction via the thromboxane receptor [111]. A study involving atopic asthmatic patients could show, however, that thromboxane receptor blockade only partially reduced bronchoconstriction in response to PGD_2_ and PGD_2_-induced bronchoconstriction potentially involves a vascular component [112]. Further, PGD_2_-DP1 signalling but not PGD_2_-DP2/CRTH2 signalling induced cough in guinea pigs by activation of vagal sensory neurons [113]. Also, in comparison to wild-type mice, DP1 KO mice subjected to OVA-induced allergic inflammation showed less mucus-containing cells in the airways as well as reduced airway hyper-reactivity [18]. In contrast, PGD_2_/CRTH2/DP2 signalling induces smooth muscle cell proliferation [104], which also contributes to airway narrowing in asthma. As a consequence, both PGD_2_ receptors, DP1 and DP2/CRTH2, have emerged as potential drug targets for the treatment of allergic asthma [22,114,115,116,117]. 

In combination with allergen exposure and genetic risk factors, lower respiratory viral infections have been associated with a higher likelyhood of developing asthma as well as disease progression and acute asthmatic exacerbations [118]. Recently, it has been shown that PGD_2_ production is elevated in viral respiratory infections and in turn promotes disease severity via DP2/CRTH2 [93]. This study also showed that hPGDS expressing immune cells were present immediately after infection while at later time points bronchial epithelial cells upregulated hPGDS and PGD_2_ production. Further, allergic inflammation is often associated with excessive eosinophil influx and activation resulting in ongoing inflammation and tissue injury. Elevated PGD_2_ levels prolong eosinophil survival via the DP1 receptor and attract more eosinophils and Th2 cells via DP2/CRTH2 from the bloodstream [48,54]. In aspirin-exacerbated respiratory disease, hPGDS-expressing eosinophils seem to contribute to elevated PGD_2_ levels as well as in allergic inflammation, which could be blocked with the hPGDS inhibitor HQL-79 [33,50]. Besides eosinophils, basophils have also been suggested as potential PGD_2_ sources in IgE-mediated inflammation [56]. As potent drivers of type-2 inflammation and their role in allergic asthma, ILC-2s have been of great interest lately. Inhibition of autocrine PGD_2_ production by human ILC-2s with hPGDS inhibitor KMN-698 abolished IL-5 and IL-13 production as well as CD25 upregulation [84]. In allergic rhinitis, hPGDS but not LPGDS expression could be detected in the nasal mucosa, while various hPGDS expressing infiltrating cells were identified [43]. Blockade of hPGDS function by treatment of antigen-challenged guinea pigs with hPGDS inhibitor TAS-204 or TFC-007 suppressed nasal blockage, nasal airway resistance and eosinophil infiltration [119,120]. Treatment with hPGDS inhibitor HQL-79 suppressed OVA-induced allergic airway inflammation in wild types and also in mice overexpressing human hPGDS [28]. HQL-79 has originally been developed as histamine receptor 1 antagonist; however, in the same study they could show that an anti-allergic effect could also be seen in histamine receptor 1 knock-out mice claiming that the beneficial effect was due to reduction of PGD_2_ synthesis. Severe asthma often involves airway remodelling including smooth muscle cell hyper-proliferation and activity. PGD_2_ promotes smooth muscle cell proliferation via DP2/CRTH2 activation and contributes to a hyper-responsive phenotype seen in asthmatic patients [103,104]. In summary, therapeutically blocking hPGDS and PGD_2_ biosynthesis could be beneficial to delay the onset of allergic asthma, reduce eosinophilic and Th2 type inflammation, alleviate symptoms including allergic rhinitis and cough as well as reduce airway remodelling.

### 4.2. Atopic Dermatitis

Atopic dermatitis is a chronic inflammatory skin disease which affects up to 20% of children and 10% of adults in industrialized countries [121]. Patients suffer from erythema, edema and crusting whereby skin barrier defects and immune hyper-activation are major components of disease development and progression [122]. In atopic dermatitis, Langerhans cells, plasmatoid and myeloid dendritic cells express hPGDS and are able to modulate PGD_2_ production upon activation with exogenous stimuli like bacterial LPS [82]. In addition, epidermal keratinocytes of atopic dermatitis patients are also able to produce LPGDS-derived PGD_2_, whereby the PGD_2_-metabolite 15d-PGJ_2_ seems to attenuate TSLP production of keratinocytes [97]. Indeed, PGD_2_ has opposing roles in skin inflammation as it has been shown to suppress inflammation via the DP receptor in the early phase while enhancing inflammation via DP2/CRTH2 receptor in the late phase in croton oil-induced dermatitis in mice [12]. In this study, transgenic hPGDS overexpressing mice showed reduced vascular leakage in the early phase but increased immune cell infiltration in the late phase thereby prolonging inflammation. Similar results were seen in the later phase of hapten-specific IgE-induced skin inflammation: PGD_2_/DP2/CRTH2 signalling resulted in lymphocyte, eosinophil and basophil infiltration as well as increased levels of macrophage chemokines which was suppressed by DP2/CRTH2 antagonist ramatroban or hPGDS inhibitor HQL-79 [16]. Therefore, blocking mast cell, dendritic cell and macrophage-derived PGD_2_ production by inhibiting hPGDS could help to reduce eosinophil, basophil and Th2 cell numbers as well as inflammatory cytokine levels in atopic dermatitis lesions. 

### 4.3. Food Allergy and Gastrointestinal Allergic Disorder

The allergic reaction to food proteins can be mediated by IgE-dependent and -independent pathways, sometimes even resulting in life-threatening anaphylactic responses [123]. Food allergies are frequent disorders in most countries and typically occur for the first time during early childhood. Allergic reactions are mostly type-2 cytokine-driven and manifest with various symptoms from skin rashes, eczema, nausea, abdominal pain, respiratory hyper-sensitivity up to anaphylactic shock [124]. Current standard of care primarily suggests to avoid food allergens and treatment of systemic anaphylactic reactions with adrenaline [125]. Specialized preventive and therapeutic options would be of substantial value for effective disease management. Maeda et al. stated that high levels of urinary PGD_2_ metabolite tetranor-PGDM can be found in patients with food allergy but not in those with other allergic diseases or healthy volunteers [41]. Further, they could see that OVA-induced intestinal allergic inflammation as well as milk-induced food allergy in mice resulted in increased urinary tetranor-PGDM, which reflected the severity of allergic symptoms and intestinal mast cell hyperplasia. Consistently, hPGDS knock-out but not LPGDS knock-out mice had reduced levels of urinary tetranor-PGDM after allergen challenge. Intestinal mast cells express high levels of the hPGDS enzyme and play a key role in IgE-mediated allergic reactions to food allergens. In contrast to the previous study, Nakamura et al. observed an increased OVA-induced intestinal allergic inflammation in mast cell-specific hPGDS knock-out mice [42]. This effect could be minimized by providing hPGDS sufficient bone marrow-derived mast cells. Hematopoietic PGDS expression in mast cells seems to be important for balancing intestinal inflammatory reactions, however, hPGDS expression in other cell types has been associated with pathogenic function. There is evidence that the TSLP/ /hPGDS/PGD_2_/DP2/CRTH2 axis may potentiate an interaction between a hPGDS^+^ Th2 subpopulation and ILC2 cells to amplify eosinophilic inflammation [126]. It was shown that TSLP-activated DCs induce hPGDS upregulation in CD4^+^ Th2 cells [86], which might contribute to increased PGD_2_ levels, activation of DP2/CRTH2+ memory T cells and enhanced ILC2 function. A pro-eosinophilic, pathogenic human DP2/CRTH2^+^/hPGDS^+^ Th2 cell subpopulation was discovered in patients with eosinophilic gastrointestinal disorder (EGID). These patients have characteristically higher numbers of DP2/CRTH2^+^/hPGDS^+^ Th2 cells, which display enhanced type-2 cytokine production thereby driving allergic eosinophilic inflammation [89]. PGD_2_/DP2/CRTH2 interaction is a strong chemotactic stimulus for eosinophils and blocking DP2/CRTH2 with specific antagonists could alleviate symptoms in eosinophilic esophagitis patients [127,128]. In summary, a reduction of PGD_2_ levels in allergic gastrointestinal inflammation could be beneficial to diminish eosinophilic inflammation, and ILC2 and Th2 cell activation. 

### 4.4. Anaphylactic Reaction

Anaphylaxis is a life-threatening condition resulting from IgE-mediated mast cell and basophil activation followed by the release of inflammatory mediators e.g., histamine and PGD_2_ into the bloodstream causing systemic inflammation [129]. From a study with 35 anaphylactic patients, the PGD_2_-derived metabolite 9α, 11β-PGF_2_ emerged as potential urinary biomarker as it correlated with the severity of the reaction [130]. However, a study in mice revealed that mast cell-derived PGD_2_ attenuates vascular hyperpermeability [15], which is characteristic for anaphylactic shock, by activation of the DP1 receptor on endothelial cells and strengthening of endothelial barrier function [131]. Additionally, hPGDS knock-out mice exhibit a stronger anaphylactic reaction which could be rescued by additional treatment with DP1 agonist BW 245c [15]. The effect of PGD_2_ in anaphylactic reactions is still inconclusive; however, the beneficial effects of PGD_2_/DP1 signalling seen in mouse models warrants further investigation. 

## 5. Patents and Clinical Studies

Several patents and patent applications for hPGDS inhibitors as therapeutic option in allergic inflammation, asthma, chronic obstructive pulmonary disease and other inflammatory diseases have been issued [132,133,134]. Regardless of more and more commercially available compounds (Table 2), only few clinical studies evaluating hPGDS inhibitor safety and efficacy can be found. In 2015, Phase I clinical trial (NCT 02397005) was initiated to evaluate tolerability and pharmacokinetics of the selective and reversible hPGDS inhibitor ZL-2102 for treatment of COPD, asthma and idiopathic pulmonary fibrosis [135]. In this clinical trial, 120 participants were enrolled to perform a randomized, double-blind, placebo-controlled study of ascending single and repeated oral doses of ZL-2102; currently the estimated study completion date is August 2019. While these results will give a first impression of the tolerability of hPGDS inhibitors in patients, considerable effort will have to be put into stratification of patients, i.e., to identify those who might benefit most from hPGDS inhibiton, for future clinical studies evaluating hPGDS inhibitors. Beyond allergic inflammation as the expected indication ofor hPGDS inhibitors, another Phase I clinical trial investigating safety and pharmacokinetics of hPGDS inhibitor TAS-205 in 23 boys with Duchenne’s muscular dystrophy could be completed successfully in 2018 [136]. Their rationale was based on studies showing increased PGD_2_ levels in myonecrotic areas presumably driving inflammation as well as an increase of urinary tetranor-PGD metabolite in Duchenne’s patients. Hematopoietic PGDS inhibitor TAS-205 was tested over various dose ranges and was deemed safe and tolerable in patients with Duchenne’s muscular dystrophy. Urinary PGD-metabolite was significantly reduced following treatment without interfering with urinary PGE_2_ metabolite levels—fostering the hypothesis that hPGDS inhibitor TAS-205 in this dosage range is well tolerated with significant biological activity in patients. 

Notably, clinical research on hPGDS inhibitors for allergic and other inflammatory disorders is still in its infancy in contrast to i.e., CRTH2/DP2 antagonists and the therapeutic potential of hPGDS inhibitors will need further exploration to confirm efficacy.

## 6. Future Perspectives

**Figure 2:**
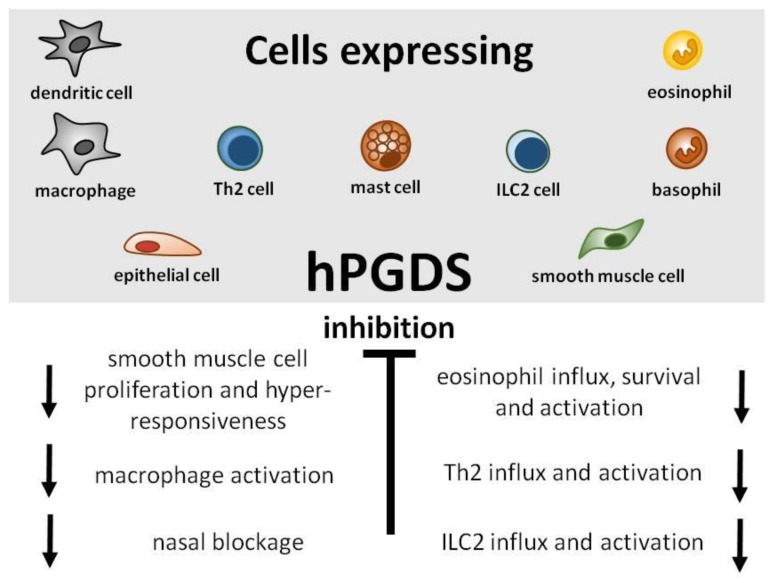
Potential target cells and beneficial effects of hPGDS inhibition in allergic inflammation. Hematopoietic PGDS expression has been reported in many cell types involved in allergic inflammation. Elevated PGD_2_ levels have been shown to induce DP2/CRTH2-mediated smooth muscle cell proliferation and hyperresponsiveness [103], macrophage activation [11], nasal blockage [10] as well as influx, activation and survival of eosinophils, ILC2s and Th2 cells. Inhibition of hPGDS-derived PGD_2_ production would target all above-mentioned pathological phenomena.

**Figure 3:**
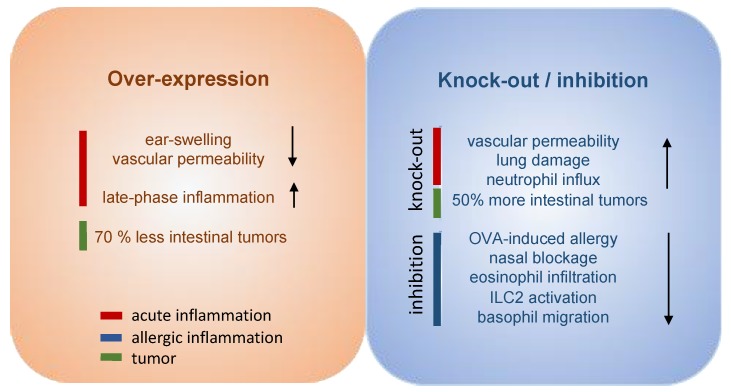
Experimental overexpression, inhibition and knock-out of hPGDS differentially modulates acute and chronic inflammation. Overexpression of transgenic hPGDS in mice resulted in reduced ear swelling and vascular permeability in the acute phase of inflammation, however, it exacerbated leukocyte influx in the late phase; In the same model, hPGDS knock-out potentiated vascular extravasation during acute skin inflammation [12]. Neutrophil influx and lung damage was more prominent in hPGDS knock-out mice [14]. In contrast, hPGDS inhibition proved to be beneficial in experimental models of allergic inflammation [28,119,120]. Interestingly, hPGDS over-expression was able to reduce the number of intestinal tumors, while hPGDS knock-out showed the opposite effect [27].

Great efforts have already been put into the development and evaluation of hPGDS inhibitors to diminish PGD_2_-related exacerbation of inflammation without interfering with LPGDS-derived PGD_2_ production important for endothelial cell function. Clinically efficient, commercially available hPGDS inhibitors would enable targeting various hPGDS-expressing cell types simultaneously and hence reduce recruitment and activation of many inflammatory cells involved in allergic progression (Figure 2). Therapeutic efficacy of hPGDS inhibitors might strongly depend on disease phenotype, i.e., the source of elevated PGD_2_ levels, target cells involved in inflammatory reaction. It has been suggested that PGD_2_ exerts a biphasic effect in inflammation, i.e anti-inflammatory effects were observed in the acute phase of inflammation, whereas pro-inflammatory DP2/CRTH2-mediated effects, i.e., chemotaxis and activation of effector cells, prevail in the late phase; this proposes timing of treatment as another variable yet to consider to successfully select target groups. Hematopoietic PGDS overexpression, knock-out and inhibition studies in mice are summarized in Figure 3 that gives an overview how systemic excess or loss of hPGDS-derived PGD_2_ influences (patho-)physiological settings. A relevant concern of hPGDS inhibition is the influence on other eicosanoids downstream of cyclooxygenases. The possibility that excess PGH_2_ will serve as precursor for other eicosanoids arises, thereby causing an imbalance in the arachidonic acid/COX pathway. Interestingly, an increased PGE_2_ to PGD_2_ ratio in patients with respiratory disorders has been associated with better prognosis [109], suggesting a positive effect of shunting excess PGH_2_ into PGE_2_ production. However, studies investigating HQL-79-induced hPGDS inhibition in mice could show that PGE_2_ and PGF_2_ levels did not change upon treatment [28,137]. Indeed, most studies that showed beneficial effects of hPGDS inhibitors were conducted in allergy models (Figure 2 and Figure 3); type-2 inflammatory disorders like allergic asthma, food allergy, EGID and atopic dermatitis involve similar effector cell types that react strongly to PGD_2_ via DP2/CRTH2 stimulation but also DP1 activation has been linked to induction of cough and eosinophil survival. Increased hPGDS expression and/or PGD_2_ production has been confirmed in patients with acute exacerbations of asthma. Viral infection upregulated hPGDS in bronchial epithelial cells and increased PGD_2_ levels were associated with aggravated respiratory inflammation [93]. As in Duchenne’s patients, increased urinary PGD metabolite has been associated with worse prognosis in Aspirin-exacerbated respiratory disease [138]. TAS-205 has been shown to efficiently reduce urinary PGD metabolite in Duchenne’s patients and would potentially be a drug candidate to investigate beneficial effects in this patient group. As already mentioned, patient stratification will be crucial to explore translational potential of novel therapeutic approaches for allergic disorders. To illustrate potential obstacles, CRTH2 inhibitors have been studied extensively in recent years both in animal models and patients, but clinical investigations are still ongoing with no approved drug on the market yet. There are still many factors to consider before clinical efficacy and translatability of hPGDS inhibitors can be explored. However, regarding the extensive number of cell types capable of producing PGD_2_ and/or being influcenced by PGD_2_ highlights hPGDS as a promising pharmaceutical target to modulate allergic respiratory inflammation. Whether the concept of inhibiting PGD_2_ biosynthesis as opposed to blocking PGD_2_ receptors is valid and clinically superior also remains to be elucidated.

## Figures and Tables

**Table 1:** Cell type specific hPGDS expression and reported PGD_2_ release.

	Cell Type	Species	hPGDS Expression	Stimuli	PGD_2_ Release	Ref.
**Parenchymal Cells**	Epithelial cells	ms hu	mRNA, protein mRNA, protein	RSV infection Unstimulated IL-13, IL-4	1.2 ng/mL 100 pg/mL 25–50 pg/mL	[43,93] [93] [105]
	Endothelial cells	hu	*LPGDS*-mRNA, protein *LPGDS*-protein	sheer stress tumor CM	150 pg/mL 600 pg/mL	[101] [102]
	Smooth muscle cells	ms	mRNA, protein	OVA lung inflammation		[103]
	Keratinocytes	hu	protein	antimycotics	8 ng/10^6^ cells	[97]
**Immune Cells**	Monocytes	hu		unstimulated	66 fmol/10^6^ cells	[67]
	Macrophages	ms	protein mRNA	LPS zymosan A -(peritoneal lavage)	20 ng/mL 800 pg/mouse	[29,73] [75]
		hu	mRNA	IL-4, HDM	400 pg/mL	[77]
		rat	protein			[11,63]
	Dendritic cells	hu	mRNA, protein	LPS, INF-γ	250 pg/10^6^ cells	[82]
		rat, h	protein			[63,83]
	Eosinophils	ms hu	mRNA, protein mRNA, protein	eotaxin/A23187 eotaxin/A23187	15 pg/2 × 10^6^ cells 15–80 pg/2 × 10^6^	[33]
		hu	mRNA, protein	Lysin-Aspirin	1.5 ng/10^5^ cells	[50]
	Neutrophils	ms	protein			[14]
	Basophils	ms hu	mRNA, protein protein	TNP-OVA (IgE) IL-3, anti IgE	700 pg/5 × 10^5^ cells 100 pg/5 × 10^5^ cells	[56] [56,57]
	Mast cells	hu rat		anti-IgE anti-IgE anti-IgE	40 ng/10^6^ cells 1 ng/10^4^ cells 13 ng/10^6^ cells	[39] [106] [39]
		ms	protein			[41,42,44]
	ILC2	hu	mRNA mRNA	IL-33, IL-25, TSLP	1.5 ng/mL	[84] [107]
	Th2 cells	hu	mRNA, protein	OKT3, KOLT-2	30 ng/5 × 10^5^	[88]
		hu	protein	PMA, ionomycin	70 pg/mL	[89]

Abbreviations: ILC—innate lymphoid cells, Th2—CD4^+^ type-2 helper T cells, ms—mouse, hu—human, IgE—Immunoglobulin E, TNP—2,4,6-Trinitrophenyl hapten, OVA—ovalbumin, LPS—lipopolysaccharide, TSLP—thymic stromal lymphopoietin, RSV—respiratory syncytial virus, CM—conditioned medium, HDM—house dust mite, PMA—phorbol 12-myristate 13-acetate, OKT-3—anti-CD3 antibody, KOLT-2—anti-CD28 antibody.

**Table 2:** hPGDS inhibitors and their cellular targets, effective concentrations and doses, and experimental settings reported in literature.

Inhibitor	Chemical Structure	Company	Cell Type/Disease	Concentration	Ref.
**HQL-79**	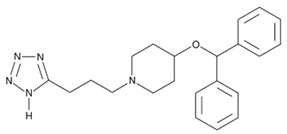	Cayman Chemicals	BMDM, eosinophils; OVA-induced lung inflammation	5–100 µM 10 mg/kg	[29,33] [28]
**TFC-007**	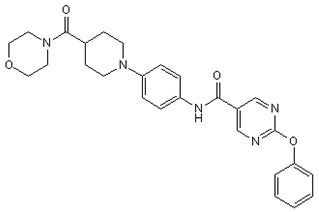	Tocris	nasal blockage in guinea pigs	30 mg/kg	[120]
**HPGDS inhibitor I**	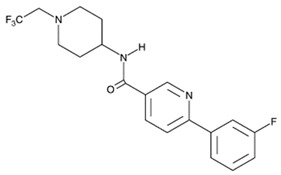	Cayman Chemicals	basophils; murine lupus model	5 mg/kg	[57]
**TAS-204**	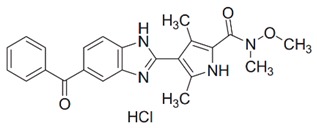	Taiho Pharmaceutical Co. Ltd.	nasal blockage in guinea pigs	15–30 mg/kg	[119]
**TAS-205**	not reported	Taiho Pharmaceutical Co. Ltd.	Duchenne‘s muscular dystrophy (Phase I)	1.67–13.33 mg/kg/dose	[136]
**KMN-698**	not reported	Sanofi Aventis (Cayman Chemicals)	ILC-2	100 nM	[84]
**ZL-2102**	not reported	Zai Lab Pty. Ltd./Sanofi	healthy males (Phase I)	5 to 750 mg	[135]

BMDM—bone marrow-derived macrophage; ILC—innate lymphoid cells; OVA—ovalbumin.
